# Gallbladder microbiota in early vertebrates provides evolutionary insights into mucosal homeostasis

**DOI:** 10.3389/fimmu.2022.1020413

**Published:** 2022-09-21

**Authors:** Li-guo Ding, Guang-kun Han, Xin-you Wang, Ru-han Sun, Yong-yao Yu, Zhen Xu

**Affiliations:** ^1^Department of Aquatic Animal Medicine, College of Fisheries, Huazhong Agricultural University, Wuhan, China; ^2^State Key Laboratory of Freshwater Ecology and Biotechnology, Institute of Hydrobiology, Chinese Academy of Sciences, Wuhan, China; ^3^Laboratory for Marine Biology and Biotechnology, Qingdao National Laboratory for Marine Science and Technology, Qingdao, China

**Keywords:** GB microbiota, evolution, mucosal immunity, IHNV infection, teleost fish

## Abstract

The gallbladder (GB) microbiota plays critical roles in mammalian metabolism and immune homeostasis, and its relationship with human disease has been extensively studied over the past decade. However, very little is known about the interplay between GB microbiota and the immune functions of teleost fish, the earliest bony vertebrate with a GB. Therefore, this study sought to investigate the composition of the teleost GB microbiota and the potential mechanisms through which it affects mucosal immunity. In our results, we found that the GB mucosa (GM) and bile bacterial community shared a similar microbiological composition with that of the gut mucosa in naïve individuals. IHNV infection induced a profound GB inflammation and disrupted their microbial homeostasis followed by a strong anti-bacterial response. Interestingly, beneficial bacteria from the Lactobacillales order showed a significant increase in the abundance of the bile microbial community, whereas the structure of the Mycoplasmatales order in the gut microbial community was markedly changed. All in all, our study characterized the structure of the GB microbial ecosystem in teleost fish, and the fish GB microbiome shared a high similarity with the gut microbiota. More importantly, our findings offer solid evidence that the teleost GB evolved immune functions to preserve its mucosal microbial homeostasis, suggesting that both the microbiota and mucosal immunity of the GB might have co-evolved in early vertebrates.

## Introduction

The gallbladder (GB) is a bladder-shaped internal mucosal organ of the digestive system of most vertebrates that consists of a surface epithelium, lamina propria, muscularis externa, and serosal layer (1). As an accessory organ of the digestive system, the GB is widely known for its function of storing and concentrating bile during digestion. Moreover, this organ acts as a reservoir and mechanical pump by modifying and circulating the bile through the enterohepatic system. Bile is a biological fluid mainly constituted by bile acids (BA), cholesterol, phospholipids, and proteins, which plays a key role in the digestive uptake of lipids and lipophilic vitamins in the intestine (2, 3). Previous studies have demonstrated that BAs possess antimicrobial properties (2, 4). Therefore, the GB environment is absent of commensal bacteria in healthy individuals, and only some GB microbes can be isolated and detected in patients with cholecystitis and cholelithiasis using culture-independent techniques (5, 6). Thus, the autochthonous microbiota of the GB has always been associated with hepatobiliary disorders. Recently, however, with the development of next-generation sequencing, the global bacterial diversity of the GM and bile in healthy individuals was first discovered in pigs and then identified in rabbits and humans (7–9). Moreover, the GB was reported to secrete mucus and participate in the construction of the biliary immune defense system (10). Additionally, bacterial infection of the GB epithelium can elicit a strong inflammatory response involving pro-inflammatory cytokine induction, massive neutrophil infiltration, and tissue injury (11). Therefore, in addition to being an important component of the digestive system, the mammalian GB is known to harbor a unique microbial community and elicit mucosal immune responses. Nevertheless, very little is known regarding the role of the GB in the mucosal microbiota homeostasis of early vertebrate species.

Among non-mammalians, teleost fish represent the oldest bony vertebrates to possess a GB (1). The teleost GB is covered by a non-keratinized epithelium, which arises from the endoderm similar to the gut mucosa (12). Previous studies have demonstrated that the fish GB epithelium has evolved absorptive and secretory functions, and the GB surface is coated with abundant mucoid substances that form a mucus layer (13, 14). Given that the teleost GB is closely connected with the foregut (FG), microbes from water and food, as well as those inhabiting the FG mucosa (FM), could reasonably pose continuous challenges to the homeostasis of the GM. Moreover, in agreement with findings in mammals, immune proteins have been identified in the bile environment of teleosts, which might play a protective role in mucosal tissues upon inflammation (15, 16). Therefore, from an evolutionary perspective, we hypothesized that the GB in both primordial and modern bony vertebrates evolved effective and similar immune mechanisms for maintaining microbial homeostasis.

To gain further insights into the microbial community structure of the bile environment of early vertebrates and assess its relationships with GB mucosal immunity, our study used the rainbow trout (*Oncorhynchus mykiss*) as a comparative immunology model. Our findings demonstrated that the autochthonous microbiota of the trout bile environment was consistent with that of the gut mucosa. As expected, a robust inflammatory response was detected in the trout GB upon infectious hematopoietic necrosis virus (IHNV) infection, which led to microbial dysbiosis followed by a strong anti-bacterial response. Interestingly, the abundance of several beneficial bacteria belonging to the Lactobacillales order increased significantly after the IHNV invasion, which might promote the anti-inflammatory response of the mucosal tissues. To the best of our knowledge, this study is the first to characterize the microbial composition of the teleost bile and identify its potential roles in GB inflammation. From an evolutionary perspective, our findings suggest that the immune functions of the GB for maintaining microbial homeostasis were already present in early vertebrates.

## Materials and methods

### Fish maintenance

Healthy rainbow trout (8-12 g) were obtained from a fish farm in Dujiangyan (Sichuan, China) and maintained in aquarium tanks with circulating water for 1 month at 16°C. Fish were fed daily with dry pellets at 1% biomass/day, and fasted two days prior to immersion infection and sampling.

### EPC cells culture and IHNV titration

ATCC CRL-2872 EPC cell line which was used for IHNV propagation was maintained in minimum essential medium (MEM, Gibco, USA) supplemented with 10% fetal bovine serum (FBS, Gibco, USA), streptomycin 100 μg/mL and penicillin (Gibco) 100 U/mL. Before IHNV infection, cells were cultured in an incubator at 28°C with 5% CO_2_ and the cell density was monitored until it reached 70 to 90% confluence. Thereafter, 10 μL of IHNV (1 × 10^7^ TCID_50_) was added to the monolayer cultures of EPC cells in a T-75 flask (Corning) supplemented with 5% FBS at 18℃. When extensive cytopathic effects (CPEs) were observed, the infected cells were collected, and followed by a gradient centrifugation (400 g for 10 min, 3,000 *g* for 10 min, and 10,000 *g* for 10 min) to remove the cells debris. The supernatant containing IHNV was filtered with a 0.22 μm PES membrane filtration (Millipore) to remove bacteria, and titrated by the Median Tissue Culture Infectious Dose (TCID_50_) assay on EPC cells following the methodology reported by Yu et al. (17).

### IHNV infection

In this study, fish were bath infected with a dose of 2 mL IHNV (1×10^8^ TCID_50_) diluted in 10 L aquatic water for 2 h at 16℃, and then migrated into the aquarium containing new aquatic water and kept for 30 days. As a control, the same number of fish were maintained in similar tanks and were exposed to the cell culture supernatant without virus. After the infection, fish were euthanized with an overdose of tricaine methanesulfonate for sampling (MS-222, Sigma).

### Microbial collection and flow cytometry analysis

Bile bacteria were isolated directly from trout GB. Mucosa-associated bacteria were collected according to a previous study with slight modification (18). Briefly, mucosa-associated tissues were collected and rinsed slightly with sterile PBS two times to remove the remaining bile or blood (BL), and then obtained by gentle blowing with sterile PBS. The collected suspension was centrifuged three times at 300 g for 5 min at 4°C to remove trout cells and debris, and further centrifuged at 16,000 *g* for 10 min followed by washing two times with sterile PBS. The obtained bacterial sedimentation was immediately frozen with liquid nitrogen and stored at -80°C. Two fish individuals were amalgamated into one sample and used for bacteria 16S rRNA gene sequencing. For flow cytometry analysis, the collected bacteria from trout GM and bile were labelled with SYTO Green-Fluorescent Nucleic Acid Stains (Invitrogen), following the manufacturer’s instructions to discriminate bacteria from debris. The analysis of stained bacteria was performed with a FACSCanto II (BD Biosciences) and FlowJo software (FlowJo LLC).

### Fluorescent *in situ* hybridization analysis

Detection of microbiota in trout GB by fluorescent *in situ* hybridization was performed as described previously with a slight modification (19). Briefly, GB cryosections (10 µm) smears from naive rainbow trout were fixed for 10 min in 4% PFA and were stained with 5’ end with Cy3-labelled EUB338 (anti-sense probe) and 5’ end with Cy3-labelled NONEUB (control sense probe complementary to EUB338) oligonucleotide probes. Hybridizations were performed at 37°C for 14 h with hybridization buffer (2 × SSC/10% formamide) containing 1 μg/mL of the labelled probes. Slides were then washed with hybridization buffer without probes followed by two more washes in washing buffer (2 × SSC) and two washes in PBS at 37°C. Nuclei were stained with DAPI (2.5 μg/mL). Images were captured using an Olympus BX53 fluorescence microscope (Olympus).

### Histology, light microscopy, and immunofluorescence microscopy studies

Trout GB were fixed in 4% neutral buffered formalin overnight at 4°, paraffin embedded, and 5 μm thick sections stained with hematoxylin-eosin (H & E) and Alcian Blue-Periodic acid-schiff (AB-PAS) as described previously (17). Images were acquired by a microscope (Olympus). For IHNV detection, sections were blocked with StartingBlock™ Blocking Buffer (Thermo), and then incubated with mouse anti-IHNV-*N* mAb (Mouse IgG isotype; 1 μg/mL; BIO-X Diagnostics) overnight at 4°C. After washing three times with PBS, the sections were incubated with Cy3-conjugated AffiniPure goat anti-mouse IgG (3 μg/mL) at room temperature for 40 min. All sections were stained with DAPI (4’, 6-diamidino-2-phenylindole; 1 μg/mL: Invitrogen) for 8 min at room temperature. Then all images were captured using an Olympus BX53 fluorescence microscope (Olympus).

### RNA extraction and qPCR analysis

Total RNA extraction from trout tissues was performed as a previous study using Trizol reagent (Invitrogen, Life Technologies), and two fish individuals were amalgamated into one sample (20). Briefly, Quality and integrity RNA analysis were used with agarose gel electrophoresis and spectrophotometry (NanoPhotometer NP 80 Touch). Then cDNA was synthesized using the Hifair III First-Strand Synthesis system (YEASEN, Shanghai, China) with 1 μg of total RNA. The synthesized cDNA was diluted to equal concentrations and used as temple for qPCR analysis according to the manufacturer’s instructions. Each tested sample was carried out on two replicate holes and followed the procedure: 95°C for 5 min, and 35 cycles of 95°C for 10 s, 60°C for 30 s. We used trout housekeeping gene elongation factor 1α (EF1α) as control and the genes expression levels were shown as 2^-ΔΔCt^ using the Pfaffl’s method (21).

### DNA extraction and PCR amplification

Total genomic DNA samples were extracted using the OMEGA Soil DNA Kit (M5635-02) (Omega Bio-Tek, Norcross, GA, USA), following the manufacturer’s instructions, and stored at -20°C prior to further analysis. The quantity and quality of extracted DNAs were measured using a NanoDrop NC2000 spectrophotometer (Thermo Fisher Scientific, Waltham, MA, USA) and agarose gel electrophoresis, respectively. PCR amplification of the total bacterial 16S rRNA genes was performed with forward primer 27F (5’-AGAGTTTGATCCTGGCTCAG-3’) and reverse primer 1492R (5’-TACGGYTACCTTGTTACGACTT-3’), bacterial V3–V4 region was performed using the forward primer 338F (5’-ACTCCTACGGGAGGCAGCA-3’) and the reverse primer 806R (5’-GGACTACHVGGGTWTCTAAT-3’). To exclude the bacterial impact from the air and water, sampling was conducted in an ultra-clean workbench, and molecular biology-grade water was used to perform DNA extractions. Thermal cycling consisted of initial denaturation at 98°C for 5 min, followed by 25 cycles consisting of denaturation at 98°C for 30 s, annealing at 53°C for 30 s, and extension at 72°C for 45 s, with a final extension of 5 min at 72°C. PCR amplicons of total bacteria were analyzed with agarose gel electrophoresis and the V3–V4 region were purified with Vazyme VAHTSTM DNA Clean Beads (Vazyme, Nanjing, China) and quantified using the Quant-iT PicoGreen dsDNA Assay Kit (Invitrogen, Carlsbad, CA, USA) for further 16S rRNA sequencing analysis.

### Sequence analysis

Amplicons were pooled in equal amounts, and pair-end 2×250 bp sequencing was performed using the Illlumina MiSeq platform with MiSeq Reagent Kit v3 at Shanghai Personal Biotechnology Co., Ltd (Shanghai, China). Sequence data analysis was mainly performed using QIIME2 and R packages (22). Sequences were quality filtered, denoised, merged and chimera removed using the DADA2 plugin (23). Taxonomy was assigned to amplicon sequence variants (ASVs) using the classify-sklearn naïve Bayes taxonomy classifier in feature-classifier plugin (24) against the SILVA Release 132 Database (25). ASV-level alpha diversity indices were calculated using the ASV table in QIIME2. Beta diversity analysis was performed to investigate the structural variation of microbial communities across samples using Jaccard metrics, and wieghted UniFrac distance metrics and visualized *via* principal coordinate analysis (PCoA). Linear discriminant analysis effect size (LEfSe) was performed to detect differentially abundant taxa across groups using the default parameters (26).

### Statistical analysis

Mean-Whitney or Kruskal-Wallis test was used for 16S rRNA sequencing statistical analysis, unpaired Student’s-*t* test, one-way analysis of variance with Bonferroni correction and Log-rank (Mantel-Cox) test (Prism version 8.0; GraphPad) were used for other experimental statistical analysis. Data are expressed as mean ± SEM. All *p* values < 0.05 were considered statistically significant.

## Results

### The teleost GM is inhabited by abundant and complex populations of commensal microbiota

**Figure 1:**
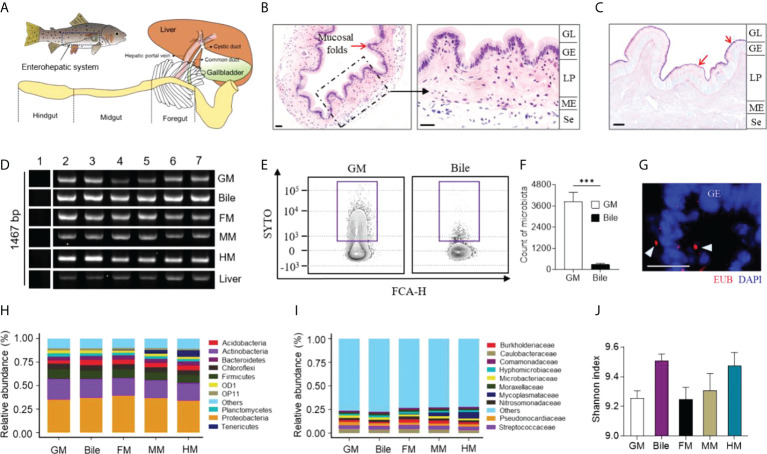
Characterization of microbiota composition in GM, bile and gut mucosa of naïve rainbow trout (*Oncorhynchus mykiss*). **(A)** Diagram of the enterohepatic system in rainbow trout. H & E **(B)** and AB-PAS **(C)** stain of GB organ obtained from naive rainbow trout. Red arrows indicate the reaction with AB-PAS. GL, GB lumen; GE, GB epithelium; LP, lamina propria; ME, muscularis externa; Se, serosa. Scale bar, 20 µm. **(D)** Real-time PCR assay analyzed the expression of 16S rRNA gene (27F and 1492R) from GM, bile, FM, MM, HM and liver of trout (*n* = 6 fish per group). Lane 1, blank control; Lane 2-7, samples from different individuals. **(E)** Representative flow cytometry staining of microbiota from trout GM (left panel) and bile (right panel) with SYTO BC Green. **(F)** Counts of positive microbiota in the GM and bile samples from naïve rainbow trout (*n* = 8 fish per group). Statistical differences were evaluated by unpaired Student’s-*t* test. **(G)** Detection of bacteria by fluorescence *in situ* hybridization in GB cryosections from naive fish. Eubacteria were detected with Cy3-EUB338 oligoprobe (red). Nuclei are stained with DAPI (blue). GE, GB epithelium. Scale bars, 20 μm. Mean relative abundance at the phylum **(H)** and family **(I)** levels of the GM, bile, FM, MM, and HM bacterial community (*n* = 8 fish per group). **(J)** Histogram showing the Shannon diversity index of GM, bile, FM, MM, and HM microbial communities from naive fish (*n* = 8 fish per group). Statistical differences were performed by Kruskal-Wallis test. Data are representative of two independent experiments (mean ± SEM). ***p < 0.001.

The teleost GB is a hepatobiliary organ for storing and secreting bile fluid, which directly connects with the liver and FG through the cystic duct in teleost fish (**Figure 1A**). Similar to other vertebrates, the teleost GM presents an extensively folded surface with regular folds and has two main layers: the epithelium, which forms the GB lumen, and the underlying layer of the lamina propria (**Figure 1B**). AB-PAS analysis identified some mucous glycoconjugates coated on the trout GM, which have an essential role in protecting the GM from the detergent effects of bile (**Figure 1C**) (27). To characterize the presence of microbiota in the trout GB, the expression patterns of the 16S rRNA gene of the GM, bile, FM, midgut mucosa (MM), hindgut mucosa (HM) and liver were analyzed *via* real-time PCR using the 27F and 1492R universal primers. As a result, amplification of the 16S rRNA gene was observed in the GM, bile, gut mucosa, and liver samples but not in the template-free blanks (**Figure 1D**). Using the SYTO BC Green-Fluorescent Nucleic Acid stain, a cell-permeant nucleic acid stain that emits a strong fluorescence signal when bound to bacterial nucleic acids, flow cytometry analysis confirmed the presence of abundant bacteria in the trout GM and low levels of bacteria in the bile (**Figures 1E, F**; samples that were not stained with SYTO BC Green are shown in **Supplementary Figure 1A**). The bacteria located in the GM were visualized *via* fluorescent *in situ* hybridization analysis (**Figure 1G**; the control probe for EUB338 is shown in **Supplementary Figure 1B**). To characterize the microbial communities of the GM and bile, 16S rRNA gene sequencing was conducted for bacterial identification. Interestingly, the trout GM, bile, and gut mucosa shared similar microbiological compositions, with most microbes belonging to the Proteobacteria, Actinobacteria, and Firmicutes phyla (**Figure 1H**). Particularly, the samples were largely dominated by the Caulobacteraceae and Streptococcaceae families (**Figure 1I**). Moreover, the Mycoplasmataceae family was only identified in the gut mucosa and its abundance was gradually decreased from the hindgut (HG) to the FG, which was consistent with the findings of a previous study (20). Therefore, the gut mucosa likely possesses unique characteristics that regulate the growth of this particular bacterial family. Moreover, α-diversity index analysis indicated that bile had higher bacterial diversity compared to the GM and gut mucosa, suggesting that a higher selective pressure likely occurs on the mucosal surface (**Figure 1J**). β-Diversity analysis showed that the bile microbiome clustered more closely with the FM and MM microbiome compared with that of the HM (**Supplementary Figure 2**).

### Inflammation in trout GB induced by IHNV infection

**Figure 2:**
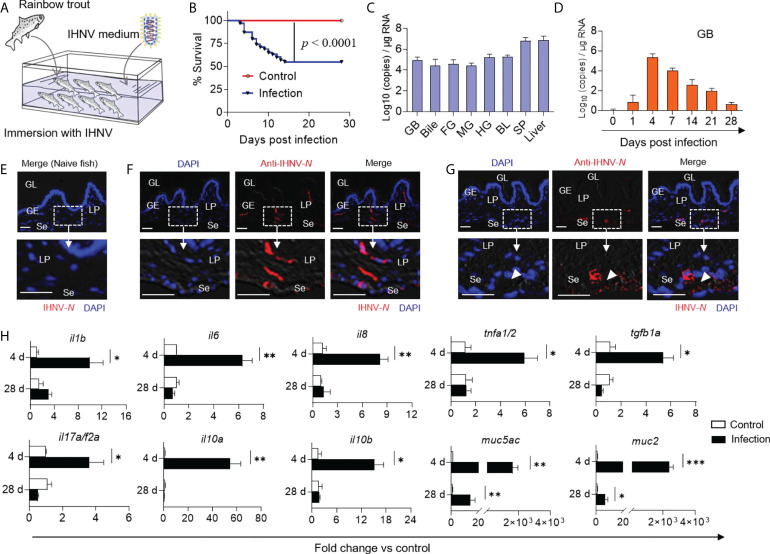
GB inflammation was induced in trout by IHNV infection. **(A)** Trout were infected with a dose of IHNV (2×10^4^ TCID_50_ per mL of water) by immersion while fish were bathed with cell culture supernatant as control. Fish were daily observed to calculate the mortality. **(B)** Cumulative survival of control and IHNV-infected fish. Statistical differences were evaluated by Log-rank (Mantel-Cox) test. **(C)** IHNV-*N* gene copies (Log_10_) were quantified using qPCR in fish GB, bile, FG, MG, HG, BL, SP, and liver collected at 4 DPI (*n* = 6 fish per group). **(D)** IHNV-*N* gene copies (Log_10_) were quantified using qPCR in fish GB organs collected at 1, 4, 7, 14, 21, and 28 DPI (*n* = 6 fish per group). Immunofluorescence staining of IHNV in GB paraffin-sections from control **(E)** and 4 days infected fish **(F, G)** (*n* = 6 fish per group). IHNV (red) were stained with an anti-IHNV-*N* mAb; Nuclei (blue) were stained with DAPI. White rectangle represents enlarged sections with some IHNV localization. White arrowhead represents artery and vein. GL, GB lumen; GE, GB epithelium; LP, lamina propria; Se, serosa. Scale bars, 20 μm. **(H)** qPCR analysis of cytokine and mucin genes in GB organ from control and IHNV-infected fish at 4 and 28 DPI. Expression levels in IHNV-infected fish were normalized to those in control fish, which were set as 1 (*n* = 8 fish per group). Statistical analysis was performed by unpaired Student’s-*t* test. Data are representative of three independent experiments (mean ± SEM). *p < 0.05, **p < 0.01, ***p < 0.001.

Previous studies have demonstrated that IHNV can invade the digestive tract and change its mucosal microbial composition (20). To evaluate the dynamic changes in the GM microbiota composition of trout upon viral infection, we developed an immersion infection model using 2 mL IHNV (2×10^8^ TCID_50_) diluted in 10 L of water (**Figure 2A**). Approximately 45% of the fish died after infection with IHNV within the first two weeks, whereas no mortality was observed in the control group during the entire experiment (**Figure 2B**). Typical IHNV symptoms (e.g., proptosis, pale gills, delayed gastric emptying, and gut emptying) were observed in infected fish at 4 days post-infection (DPI), whereas no adverse effects were observed in the control group (**Supplementary Figures 3A, B**). As expected, the results of quantitative real-time PCR (qPCR) indicated that the infected fish harbored a high viral load in the GB, bile, FG, midgut (MG), HG, BL, and spleen (SP), as well as the liver (**Figure 2C**). However, the viral loads in the GB decreased with infection time until reaching near pre-stress levels at 28 DPI (**Figure 2D**). Using an anti-IHNV-*N* monoclonal antibody (isotype-matched control antibody, as shown in **Supplementary Figure 1C**), immunofluorescence analysis confirmed that the virus was not present in the naïve fish group but was widely present in the lamina propria and serosa of the 4 DPI group (**Figures 2E, F**). Moreover, the virus was also confined to large clusters around the arteries and veins of the trout GB (**Figure 2G**), indicating that the IHNV of GB was mainly transferred from the BL through BL circulation. Additionally, the qPCR results showed that the intensity of the immune reaction of some key anti-viral genes such as *stat1*, *mx1*, *lgp2*, *tirm25*, *rag1*, *ifnar*, and *mda5* was positively related to the abundance of IHNV. Particularly, the intensity of the immune response of the GB reached its maximum at 4 DPI (**Supplementary Figure 4**). Interestingly, the pro-inflammatory cytokines *il1b*, *il6*, *il8*, *tnfa1/2*, *tgfb1a*, and *il17a/f2a*, as well as the anti-inflammatory cytokines *il10a* and *il10b*, were significantly upregulated (2.43- to 73.24-fold relative to control fish) at 4 DPI compared to the controls, and the inflammatory mediators strongly upregulated the expression levels of mucin genes such as *muc5ac* and *muc2* (1036.45- to 2919.45-fold relative to control fish) in the GB (10). At 28 DPI, however, the transcription of the immune genes returned to normal levels (**Figure 2H**; the primer sequences are shown in **Supplementary Table 1**). Taken together, our findings demonstrated that IHNV can invade the GB organ through BL circulation, and inducing pro-inflammatory responses that might be associated with microbial homeostasis.

### IHNV infection induces a profound anti-bacterial response in the trout GB

**Figure 3:**
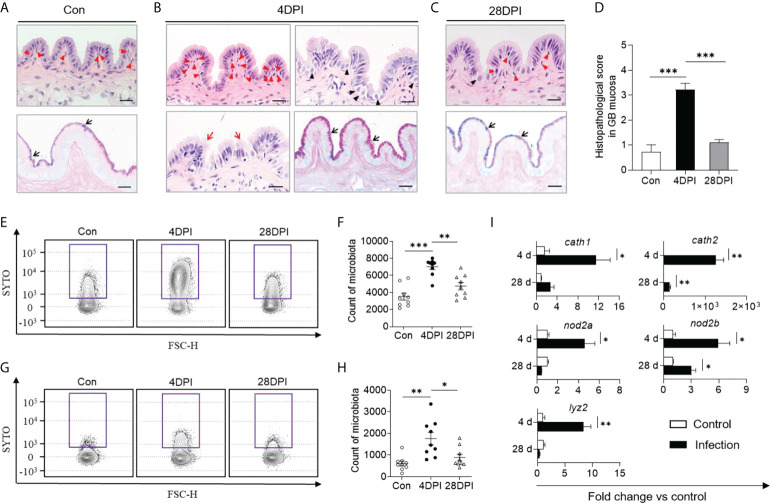
Anti-bacterial response occurred in GB following IHNV infection. Histological examination (H & E and AB-PAS) of GB organ from control (Con, **A**), and IHNV-infected fish at 4 DPI **(B)**, and 28 DPI **(C)**. Red arrowheads indicate lymphocytic cells; Black arrowheads indicate distended columnar cells with excessive mucoid secretion; Red arrows represent disrupted mucosal epithelium with loss of continuity; Black arrows indicate the reaction with AB-PAS. Scale bars, 20 μm. **(D)** Pathology score of GB from control and IHNV-infected fish at 4 DPI and 28 DPI (*n* = 6). Statistical differences were evaluated by One-way ANOVA. Flow cytometry analysis microbiota of GM **(E)** and bile **(G)** samples stained with SYTO BC Green from control and IHNV-infected fish at 4 and 28 DPI. Counts of positive microbiota in the GM **(F)** and bile **(H)** samples from control and IHNV-infected fish at 4 and 28 DPI (*n* = 9 fish per group). Statistical differences were evaluated by One-way ANOVA. **(I)** qPCR analysis of antimicrobial peptides (AMP) genes in GB organ from control and IHNV-infected fish at 4 and 28 DPI. Expression levels in IHNV-infected fish were normalized to those in control fish, which were set as 1 (*n* = 8 fish per group). Statistical analysis is performed by unpaired Student’s-*t* test. Data are representative of at least two independent experiments (mean ± SEM). *p < 0.05, **p < 0.01, ***p < 0.001.

In mammals, cholecystitis is often accompanied by histological alterations (28). Therefore, we next sought to determine whether similar lesions occurred in the trout GB following IHNV-triggered inflammation. Consistent with the high viral loads in the trout GB, some histological changes including lymphocytic infiltration, distended columnar cells with excessive mucoid secretion, disrupted mucosal epithelium with loss of continuity, and a stronger reaction to the AB-PAS stain were detected in the trout GM at 4 DPI, after which the tissues returned to normal at 28 DPI (**Figures 3A–D**; the scoring system for histopathological evaluation is summarized in **Supplementary Table 2**). Using flow cytometry analysis, we found that the bacterial structure and abundance were significantly increased at 4 DPI in the GM and bile samples compared with the control group (**Figures 3E–H**). Furthermore, a strong increase in the transcript levels of *cath2* (~1.26×10^3^-fold relative to control fish) and moderate increases in the transcript levels of *cath1*, *lyz2*, *nod2a*, and *nod2b* (2.39- to 17.77-fold relative to control fish) were detected at this time point, which has been associated with microbial dysbiosis in mammals (**Figure 3I**) (29). In contrast, the bacterial structure and abundance as well as the anti-bacterial gene expression levels returned to normal at 28 DPI (**Figures 3E–I**; the primer sequences are summarized in **Supplementary Table 1**). Collectively, our findings demonstrated that IHNV infection might induce bacterial secondary infection, which can lead to histological changes in the trout GB.

### IHNV infection leads to bacterial dysbiosis in the trout GM, bile, and gut mucosa

**Figure 4:**
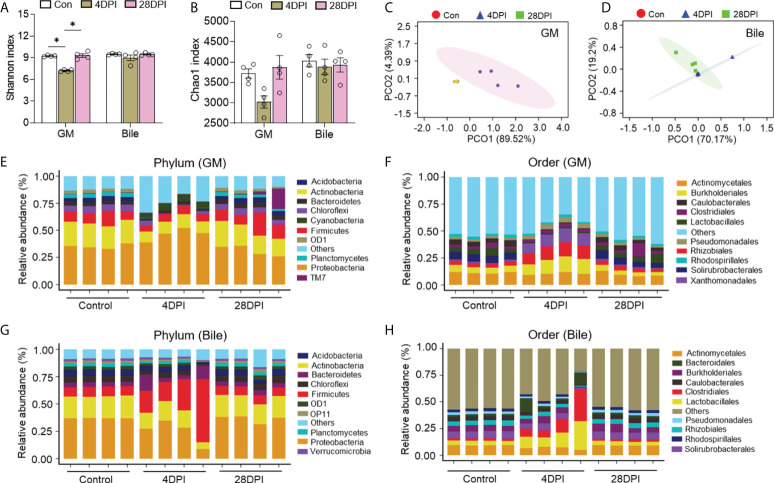
IHNV invasion caused the changes of microbial diversity and composition in trout GM and bile. Shannon **(A)** and Chao1 **(B)** diversity index of the GM and bile microbiota community from control fish and trout infected with IHNV at 4 and 28 DPI (*n* = 8 fish per group). Statistical differences were evaluated by Kruskal-Wallis test. PCoA with weighted UniFrac distance matrix for the GM **(C)** and bile **(D)** microbiota community from control fish and trout infected with IHNV at 4 and 28 DPI. Each color represents one sample (*n* = 8 fish per group). Relative abundance at the phylum **(E)** and order **(F)** levels of GM microbiota community from control and IHNV-infected fish at 4 and 28 DPI (*n* = 8 fish per group). Relative abundance at the phylum **(G)** and order **(H)** levels of bile microbiota community from control and IHNV-infected fish at 4 and 28 DPI (*n* = 8 fish per group). Data are representative of two independent experiments (mean ± SEM). *p < 0.05.

To assess the effects of IHNV infection on the microbial composition of the trout GM, bile, and gut mucosa, microbial samples from control and IHNV-infected trout were collected at 4 and 28 DPI for 16S rRNA sequencing analysis. The amplicon libraries ranged in size from 67,554 to 112,259 reads (**Supplementary Table 3**). A total of 67,554 sequences were used for normalizing the inter-sample variability. Unlike the changes in bacterial structure and abundance discussed above, microbial diversity decreased significantly in the GM as well as in the gut mucosa at 4 DPI. In contrast, the bacterial community composition of the bile samples exhibited low variation according to the Shannon and Chao1 diversity indices. At 28 DPI, no significant changes in α-diversity indices were detected in the GM, bile, and gut mucosa compared with the control group (**Figures 4A, B**; **Supplementary Figures 5A, B**). Next, PCoA based on the weighted UniFrac distance matrix was conducted to categorize the different bacterial groups in the GM, bile, and gut mucosa samples. Our findings indicated that the microbiome pattern of the control group was different from that of the 4 DPI group, but similar to that of the 28 DPI groups, especially in the GM, MM, and HM samples (**Figures 4C, D**; **Supplementary Figures 5C–E**). To further analyze the composition of the microbiota of each group, we classified the phylum and order of the microbial sequences from control and IHNV-infected trout at 4 and 28 DPI. At the phylum level, we observed an increase in the abundance of Proteobacteria (35.2% control versus 46.4% 4 DPI) as a result of IHNV infection and a decrease in Firmicutes abundance (10.5% control versus 6.7% 4 DPI) in GM. However, in the bile microbial community, the abundance of Proteobacteria decreased from 37.0% in the control group to 27.8% in the 4 DPI group, whereas the Firmicutes abundance (8.7% control versus 31.0% 4 DPI) was higher in the 4 DPI group compared with that of the control group (**Figures 4E, G**). At the order level, IHNV infection resulted in significant reductions in the abundance of Caulobacterales (4.8% control versus 0.6% 4 DPI) and beneficial short-chain fatty acids producers such as Lactobacillales in the GM (5.0% control versus 1.0% 4 DPI), coupled with marked increases in the abundance of the orders Burkholderiales (5.9% control versus 12.8% 4 DPI), Rhizobiales (3.8% control versus 12.1% 4 DPI), and Xanthomonadales (1.1% control versus 11.2% 4 DPI) in the 4 DPI group (**Figure 4F**). In the bile microbial community, an increased abundance of Clostridiales (2.3% control versus 14.4% 4 DPI) and Lactobacillales (4.1% control versus 15.0% 4 DPI) was detected upon IHNV infection (**Figure 4H**). At the phylum level, the microbial composition of the gut mucosa of the control groups was dominated by Proteobacteria (39.2% FM, 36.9% MM, 33.9% HM), followed by Actinobacteria (18.6% FM, 18.6% MM, 18.6% HM) and Firmicutes (9.6% FM, 8.5% MM, 8.6% HM). In response to IHNV infection, the community of the FM and MM shifted and became dominated by Proteobacteria (29.1% FM, 8.2% MM), Tenericutes (22.8% FM, 70.0% MM), and Actinobacteria (15.9% FM, 0.40% MM). Proteobacteria (23.3%), Actinobacteria (6.9%), and Firmicutes (4.4%) were still prevalent in HM at 4 DPI (**Supplementary Figures 6A–C**). Furthermore, sequences belonging to the order Mycoplasmatales were more abundant in the FM and MM samples from the 4 DPI group (22.8% FM, 70.0% MM) compared with the samples from the control group (0.5% FM, 3.8% MM). In the HM samples, the abundance of the members of the order Mycoplasmatales decreased from 6.7% in the control group to 0.9% in the 4 DPI group, suggesting a microbial transfer might occur in the gut mucosa during IHNV infection (**Supplementary Figures 6D–F**). At 28 DPI, the bacterial community composition across all the samples showed minor differences compared with the control fish (**Figures 4E–H**; **Supplementary Figure 6**).

### IHNV infection exerts different selective pressures on the GM, bile, and gut mucosa bacteria

**Figure 5:**
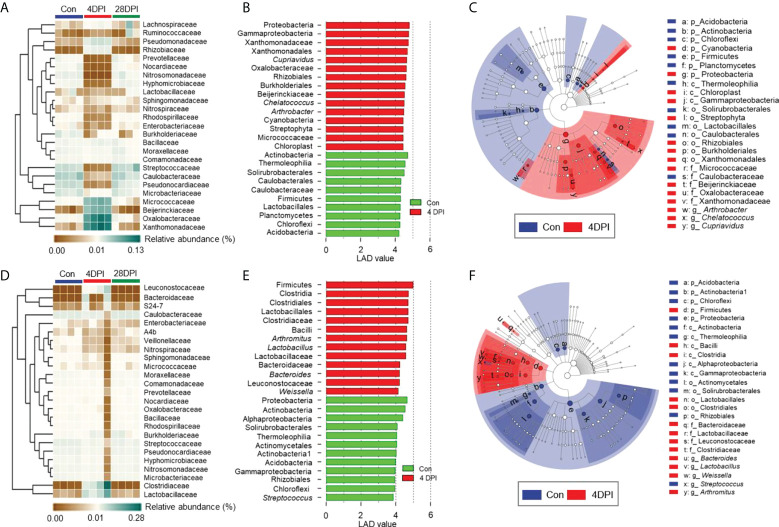
Differential bacterial taxa between the control and 4 DPI groups in GM and bile. Heat map showing the hierarchical clustering results for the abundance of top 25 bacterial family in the GM **(A)** and bile **(D)** from control fish and trout infected with IHNV at 4 and 28 DPI (*n* = 8 fish per group). Each column represents one sample. Bar chart of the log-transformed LDA score of bacterial taxa found to be significantly associated with control fish and trout infected with IHNV at 4 DPI in GM **(B)** and bile **(E)** by LEfSe (*p* < 0.05) (*n* = 8 fish per group). Cladogram representation of LDA analysis in B and E showing the phylogenetic relationships among the bacterial taxa found to be significantly associated with control fish and trout infected with IHNV at 4 DPI in GM **(C)** and bile **(F)** by LEfSe (*p* < 0.05) (*n* = 8 fish per group). Data are representative of two independent experiments.

**Figure 6:**
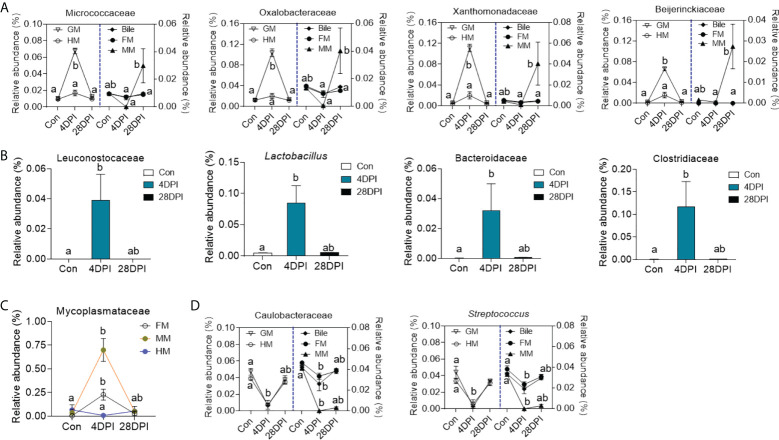
Dynamic alteration of microbial microbes in GM, bile and gut mucosa upon viral infection. **(A)** Line chart showing the representative abundance from top 50 bacterial families in GM, bile, FM, MM, and HM from control and IHNV-infected fish at 4 and 28 DPI (*n* = 8 fish per group). Statistical differences were evaluated by Kruskal-Wallis test. **(B)** Histogram showing the representative abundance of bacterial taxa in bile from control and IHNV-infected fish at 4 and 28 DPI (*n* = 8 fish per group). Statistical differences were evaluated by Kruskal-Wallis test. **(C)** Line chart showing the representative abundance of Mycoplasmataceae family in FM, MM, and HM from control and IHNV-infected fish at 4 and 28 DPI (*n* = 8 fish per group). Statistical differences were evaluated by Kruskal-Wallis test. **(D)** Line chart showing the representative abundance of bacterial taxa in GM, bile, FM, MM, and HM from control and IHNV-infected fish at 4 and 28 DPI (*n* = 8 fish per group). Statistical differences were evaluated by Kruskal-Wallis test. Data are representative of two independent experiments (mean ± SEM). The different lowercase letter represent significant difference (*p* < 0.05) exist in these groups within each sample.

Details on the changes in the GM and bile microbial community at the family level for the top 25 most abundant operational taxonomic units (OTUs) were visualized in heat maps. The microbial structure of the control and 4 DPI groups exhibited significant differences in relative abundance at varying levels (**Figures 5A, D**). Therefore, linear discriminant analysis coupled with effect size (LEfSe) analysis was conducted to compare the microbial structures of these two groups. As shown in **Figures 5B, C**, the Micrococcaceae, Oxalobacteraceae, Xanthomonadaceae, and Beijerinckiaceae families showed a significant increase in the GM microbial community after viral infection, whereas no significant differences in bacterial communities were observed in the bile and gut mucosa samples between control and 4 DPI groups (**Figures 5B, C**; **Figure 6A**). Moreover, bacterial taxa of Leuconostocaceae, *Lactobacillus*, Bacteroidaceae, and Clostridiaceae were markedly enriched in the bile bacterial community from the top 25 taxonomic features of the 4 DPI group but showed no significant change in other samples (**Figures 5E, F**; **Figure 6B**). The Mycoplasmataceae family, which was only detected in the gut mucosa, showed a significant increase in the FM and MM after IHNV infection, but decreased in the HM, suggesting a potential microbial shift in the gut mucosa during IHNV infection (**Figure 6C**). Additionally, the abundances of Caulobacteraceae and *Streptococcus*, which were the dominant bacteria in naïve individuals, decreased significantly across all samples at 4 DPI when compared with that of the control group, suggesting that these bacteria failed to compete with other bacteria and/or were particularly vulnerable in the inflammatory environment (**Figure 6D**). Taken together, our findings suggest that IHNV infection might exert various selective pressures on different mucosal microbiota, resulting in unique variations in the microbiome of the GM, bile, and gut mucosa.

## Discussion

The GB of vertebrates is an auxiliary digestive organ that is anatomically conserved across species, and the bile inside the GB plays a key role in host metabolism and health (1, 9). This organ was widely thought to be sterile under normal conditions because of the physical and chemical features of bile and its antimicrobial activity (2, 4). Nevertheless, recent studies identified an unexpectedly rich bacterial community in the biliary tract of healthy tetrapods *via* 16S rRNA gene sequencing techniques (7–9). Still, very little is known regarding the role of the mucosal microbiota of the GM of early vertebrates. To the best of our knowledge, this study is the first to demonstrate that the GM of teleost fish also harbored an autochthonous microbial community, in addition to providing insights into the potential interplay between GB microbiota and mucosal immunity.

By conducting 16S rRNA analysis, our study demonstrated that members of the Proteobacteria, Firmicutes, and Actinobacteria phyla were the dominant native microbiota in the GB, which was consistent with the microbial structure of the teleost gut mucosa (30). Moreover, bacterial composition analysis at the family level showed that gram-negative bacteria occupied most of the bacterial community, thus confirming that gram-negative bacteria were inherently more resistant to bile than gram-positive bacteria (2). Furthermore, the bacterial structure and abundance of the GM was higher than that of the bile samples, but the bacterial diversity was lower than that of the bile bacterial community, indicating that some specific microbiota from the bile could colonize the GM and proliferate. In a recent study, the human duodenum and GB microbiome were closely clustered based on the Bray-Curtis dissimilarity matrix, suggesting that the GB microbiome has a close relationship with that of the small intestine (31). In teleost fish, the gut is roughly divided into three segments: the FG, MG, and HG (20). The FG and HG constitute more than two-thirds of the length of the trout intestine, and they are the main segments involved in lipid digestion and absorption, which is analogous to the functions of the anterior small intestine in humans (32–35). Our findings demonstrated that the microbial diversity of the FM and MM exhibited lower richness than that of the HM, suggesting a higher selective pressure in FM and MM than HM. β-diversity analysis showed that the bile microbiome clustered more closely with the FM and MM microbiome compared with that of the HM. According to a previous study, bile microorganisms likely originate from excretions from the liver or ascend from the intestine (36). Therefore, we speculate that the bile microbiota might arise from the FM and MM in teleost fish, which then colonize the GM.

In mammals, viral infections are associated with acute acalculous cholecystitis, which can lead to secondary bacterial infection in the bile environment (37, 38). To investigate whether viral infection can generate a GB immune response in teleost fish and assess the changes in the microbial structure of the bile environment, we established an infection model by immersion with IHNV, which is a fatal Novirhabdovirus that affects salmonid species including rainbow trout. Importantly, we have previously demonstrated that IHNV can invade the internal organs of rainbow trout, including the gut and swim bladder (17, 20). Using our previously reported immersion infection approach, our study demonstrated that IHNV can reach the GB organ through BL circulation and induce high expression levels of the *stat1*, *mx1*, *lgp2*, *tirm25*, *rag1*, *ifnar*, and *mda5* genes, all of which have been associated with antiviral immunity (39–43). Notably, the expression levels of pro-inflammatory cytokines (*il1b*, *il6*, *il8*, *tnfa1/2*, *tgfb1a*, and *il17a/f2a*) as well as anti-inflammatory cytokines (*il10a* and *il10b*) were both upregulated at 4 DPI and recovered at 28 DPI in the GB. These effects were consistent with the variations in the viral loads with infection time, suggesting that IHNV infection induced a strong inflammatory reaction in the trout GB. In mammals, inflammatory mediators can positively regulate the expression of several mucin genes such as *muc2* and *muc5ac*, and therefore participate in the biliary immune defense system (10). Similar to previous reports in mammals, our study demonstrated that these two mucin genes were significantly upregulated in the trout GB at 4 DPI, which was accompanied by some histological changes such as distended columnar cells with excessive mucoid secretion and a stronger reaction to AB-PAS stain (28). Moreover, a significant increase in the microbiome structure and abundance followed by anti-bacterial responses were observed in the GM of IHNV-infected trout, indicating that IHNV infection disrupted the GM microbial homeostasis and induced immune responses in the GB.

Specifically, the abundance of the Proteobacteria phylum in the GM microbial community was significantly increased after IHNV infection, and this phylum is known to encompass multiple pathogens with pro-inflammatory properties (44). For example, Xanthomonadaceae, Burkholderiales, and Rhizobiales, which were the primary contributors to the Proteobacteria abundance at 4 d after IHNV infection, also increased significantly in mammals after infection with certain viruses (45–47). Therefore, the aforementioned bacteria might induce a dramatic inflammatory and anti-bacterial response in the GB, resulting in GB tissue damage in IHNV-infected trout. Conversely, IHNV infection reduced the abundance of Caulobacteraceae and *Streptococcus*, which were the predominant members of the Proteobacteria and Firmicutes phyla in naïve trout, respectively. Caulobacterales have been demonstrated to have a negative correlation with the increased expression levels of *tnfa* and *il6* transcripts while positively associated with *il10* expression, but the presence of *Streptococcus* in the digestive tract can trigger the production of pro-inflammatory cytokines (48, 49). Interestingly, *Streptococcus* abundance decreased in the bile bacterial community as well, whereas the abundance of other lactic acid bacteria such as *Lactobacillus* and Leuconostocaceae increased significantly after IHNV infection. Previous studies have shown that *Lactobacillus*, a beneficial microorganism, plays a key role in modulating dysbiosis such as inhibiting the growth of some *Streptococcus* strains and prompting the production of the anti-inflammatory cytokine *il10*, suggesting that the reduced abundance of *Streptococcus* in GM and bile bacterial community might be attributed to the increased abundance of *Lactobacillus* (50, 51). Additionally, the abundances of Bacteroidaceae and Clostridiaceae, which can promote the conversion of cholic acid (i.e., a primary BA) into deoxycholic acid (i.e., a secondary BA), also increased in the bile bacterial community, suggesting that the bile bacteria community might also be involved in the metabolism of BA (52, 53). Taken together, our findings indicated that the teleost GB has evolved an immune strategy to prevent and counteract mucosal microbial dysbiosis and the bile microbiota might contribute to maintaining the microecological balance in teleost fish.

Previous studies have reported that the pH of the bile is nearly neutral (pH = 7) in the mammalian GB, but as bile is discharged into the duodenum and mixed with chyme from the stomach, the pH in the gut mucus decreases to 5.2 (54). Therefore, the gut microbiome not only faces selective pressure from bile components but also from the gastric fluids when compared with that of the GM. In our study, we found abundant food blocked in the trout’s stomach, which was accompanied by massive ascites after IHNV infection for 4 d. In turn, this decreased the entrance of the chyme and gastric fluid mixture into the intestine, resulting in gut emptying followed by bile delays in the GB. Therefore, bacterial contact between the gut mucosa and GM is limited and a different selective pressure in these two regions would exert various alterations on the symbiotic microorganisms of the GM and gut mucosa. In the gut bacterial community, Mycoplasmatales exhibited the most obvious change in the FM and MM, whereas the GB bile environment remained largely unaltered. A previous study reported that this bacterium was highly sensitive to bile components (55). Thus, we speculated that the increase in Mycoplasmatales abundance was mainly driven by changes in the bile components in the FM and MM. In the HM bacterial community, microbial diversity, as well as the abundance of most bacteria, all decreased at 4 DPI compared with the control group, indicating that the HM bacterial loads decreased following IHNV infection. The HG generated the strongest immune response after infection or immunization, which was likely triggered to limit bacterial growth (56).

In conclusion, our study elucidated previously unrecognized autochthonous microbial communities in the teleost GM, which had a similar composition to that of the gut mucosa. IHNV infection induced inflammation of the trout GB inflammation, which was accompanied by severe bacterial dysbiosis in the GM. In turn, this resulted in secondary infection by opportunistic pathogens and strong anti-bacterial responses. More importantly, we discovered that the abundance of members of the Firmicutes phylum, many of which are known to possess anti-inflammatory functions, increased significantly after infection, suggesting that the teleost bile microbiota likely evolved to play a key role in maintaining the hemostasis of the GB mucosa (57). Collectively, our results suggest that the microbiota and mucosal immunity of the GB might have co-evolved in early vertebrates.

## Data availability statement

The datasets presented in this study can be found in online repositories. The names of the repository/repositories and accession number(s) can be found in the article/**Supplementary Material**.

## Ethics statement

The animal study was reviewed and approved by Animal procedures were approved by the Animal Experiment Committee of Institute of Hydrobiology, Chinese Academy of Sciences.

## Author contributions

L-GD designed and conducted the study as well as bioinformatics analysis and contributed to the writing of the original manuscript. G-KH contributed to DNA extraction. X-YW and R-HS contributed to qPCR analysis. L-GD, ZX, and Y-YY contributed to writing of the final manuscript. All authors contributed to the article and approved the submitted version.

## Funding

This work was supported by grants from National Natural Science Foundation of China (32225050, 32073001 and 31873045) to ZX, the National Natural Science Foundation of China (3210210315) and the State Key Laboratory of Freshwater Ecology and Biotechnology (2022FB13) to Y-YY.

## Acknowledgments

We thank Hong Liu of Shenzhen Academy of Inspection and Quarantine Sciences for providing the IHNV strain (JL14-2814, originally isolated from juvenile rainbow trout); Xue-qin Liu of College of Fisheries of Huazhong Agricultural University (Wuhan, China) for the ATCC CRL-2872 EPC cell line.

## Conflict of interest

The authors declare that the research was conducted in the absence of any commercial or financial relationships that could be construed as a potential conflict of interest.

## Publisher’s note

All claims expressed in this article are solely those of the authors and do not necessarily represent those of their affiliated organizations, or those of the publisher, the editors and the reviewers. Any product that may be evaluated in this article, or claim that may be made by its manufacturer, is not guaranteed or endorsed by the publisher.
