# Supplementary material for: Gallbladder microbiota in early vertebrates provides evolutionary insights into mucosal homeostasis
Source: Front Immunol. 2022 Sep 21;13:1020413. doi: 10.3389/fimmu.2022.1020413 (PMC9532620; doi:10.3389/fimmu.2022.1020413)
Supplement: Supplementary file 1 [file DataSheet_1.docx]

**Gallbladder microbiota in early vertebrates provides evolutionary insights into mucosal homeostasis**

**Li-guo Ding^1^, Guang-kun Han^1^, Xin-you Wang^1^, Ru-han Sun^1^, Yong-yao Yu^1^, Zhen Xu^2,3*^**

^1^ Department of Aquatic Animal Medicine, College of Fisheries, Huazhong Agricultural University, Wuhan, Hubei 430070, China

^2^ State Key Laboratory of Freshwater Ecology and Biotechnology, Institute of Hydrobiology, Chinese Academy of Sciences, Wuhan 430072, China

^3^ Laboratory for Marine Biology and Biotechnology, Qingdao National Laboratory for Marine Science and Technology, Qingdao, 266071, China

^*^Corresponding Author: zhenxu@ihb.ac.cn.

**Supplementary materials**


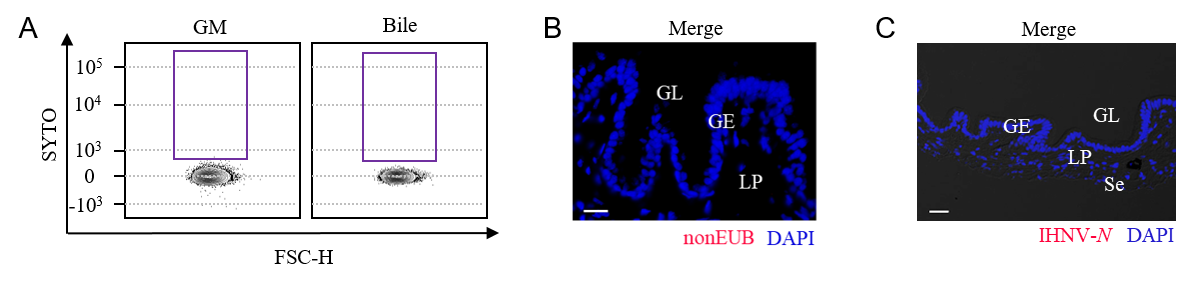


**FIGURE** **S1** | Negative stain used in this study. (**A**) Flow cytometry analysis of GM (left panel) and bile (right panel) samples without staining of SYTO BC Green from naive fish. (**B**) A control probe for EUB338 (Cy3-labelled NONEUB) was used to stain GB cryosections from naive rainbow trout by fluorescence *in situ* hybridization. Nuclei are stained with DAPI (blue). GL, GB lumen; GE, GB epithelium; LP, lamina propria. Scale bar, 20 μm. (**C**) Differential interference contrast images of GB paraffin-sections from trout infected with IHNV at 4 DPI, with merged staining of isotype control antibody for anti-IHNV-*N* mAb (red). Nuclei (blue) are stained with DAPI. GL, GB lumen; GE, GB epithelium; LP, lamina propria; Se, serosa. Scale bars, 20 µm.


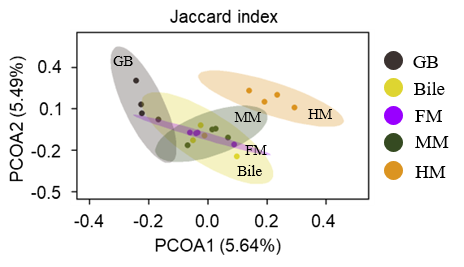


**FIGURE** **S2** | Principal coordinate analysis (PCoA) with Jaccard distance for the GM, bile, FM, MM, and HM microbiota community from control fish. Each color represents one sample (*n* = 8 fish per group). Data are representative of two independent experiments.


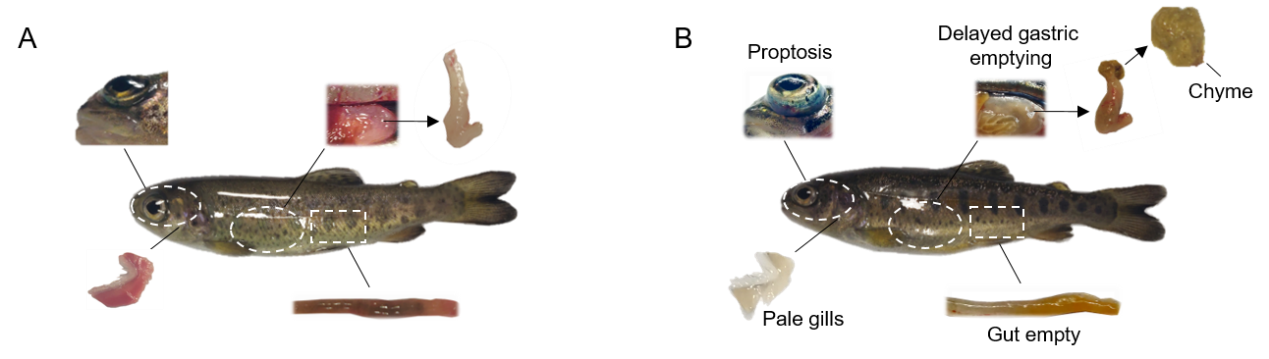


**FIGURE** **S3** | (**A**) Representative tissue morphological features from fish that bathed with cell culture supernatant for 4 days. (**B**) Typical symptom such as proptosis, pale gills, delayed gastric emptying, and gut emptying in 4-day infected fish with IHNV.


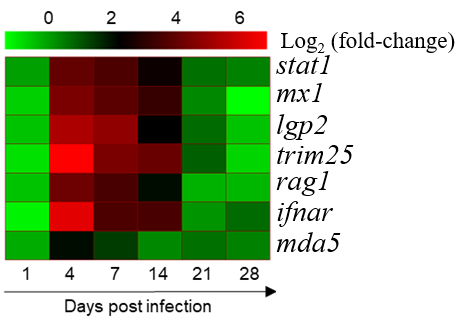


**FIGURE** **S4** | Heat map demonstrated results from qPCR of mRNAs for selected anti-viral genes in IHNV-infected fish versus control fish measured on days 1, 4, 7, 14, 21, 28 post infection in GB of rainbow trout (*n* = 6 fish). Color value, log_2_ (fold change). Data are representative of three independent experiments.


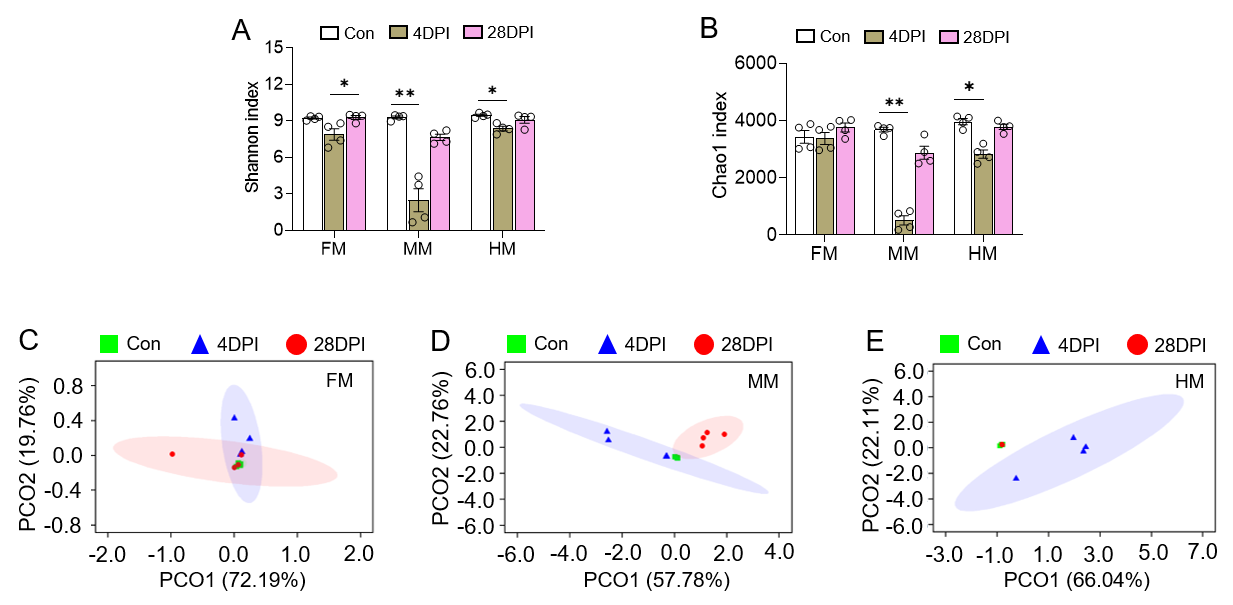


**FIGURE** **S5** | Bacterial diversity in gut mucosa from control and IHNV- infected fish. Shannon (**A**) and Chao1 (**B**) diversity index of the FM, MM, and HM microbiota community from control fish and trout infected with IHNV at 4 and 28 DPI (*n* = 8 fish per group). Statistical differences were evaluated by Kruskal-Wallis test. Principal coordinate analysis (PCoA) with weighted UniFrac distance matrix for the FM (**C**), MM (**D**), and HM (**E**) microbiota community from control fish and trout infected with IHNV at 4 and 28 DPI. Each color represents one sample (*n* = 8 fish per group). Data are representative of two independent experiments (mean ± SEM). * *p* < 0.05, ** *p* < 0.01.


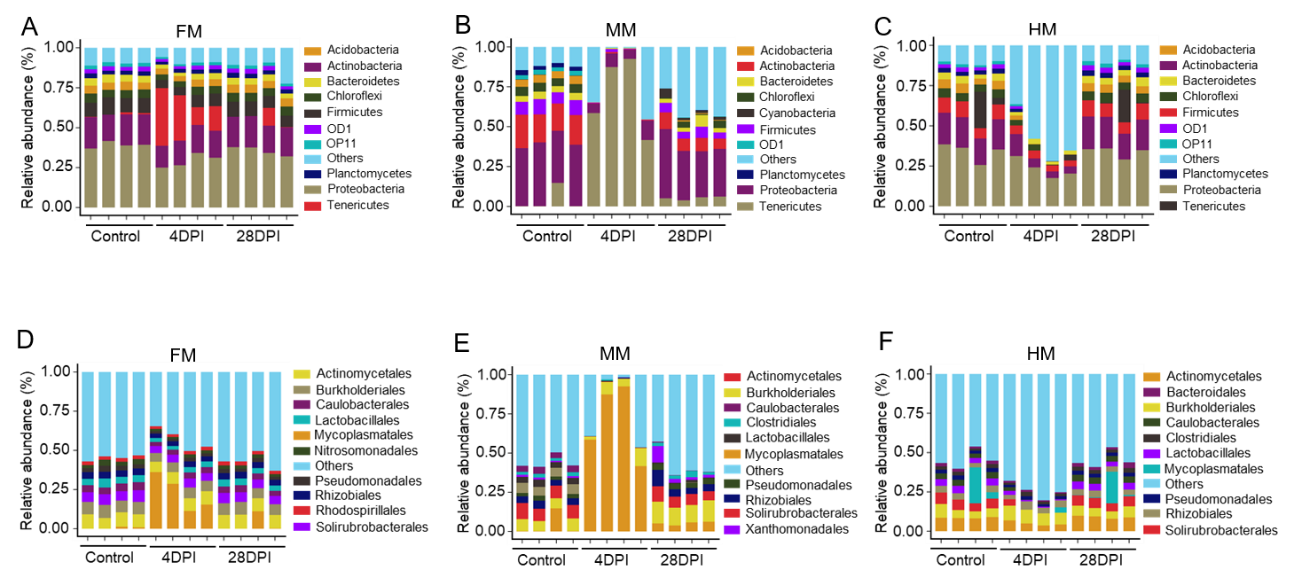


**FIGURE** **S6** | Differences in bacteria composition among control, 4 DPI, and 28 DPI group in gut mucosa. Relative abundance at the phylum levels of FM (**A**), MM (**B**), and HM (**C**) microbiota community from control and IHNV-infected fish at 4 and 28 DPI (*n* = 8 fish per group). Relative abundance at the order levels of FM (**D**), MM (**E**), and HM (**F**) microbiota community from control and IHNV-infected fish at 4 and 28 DPI (*n* = 8 fish per group). Data are representative of two independent experiments.

Table S1 Primer sequences used for qPCR.

| Primer name | Primer sequence (5’-3’) | Accession number |
| --- | --- | --- |
| EF1α-F | GGCAAGCCCATGTGTGTGGA | NM_001124339 |
| EF1α-R | ACCACCCGAGGGACATCCTG |  |
| IHNV-*N*-F | TTAACTTCAACGCCAACAGG | NP_042676.1 |
| IHNV-*N*-R | TCGGACAGGTTGATGAGAATG |  |
| STAT1-F | CTCATCCCCTGGACCAAGTT | NP_001118179.1 |
| STAT1-R | TTATTGTAGCCCTCCACCCA |  |
| MX1-F | GATGCTGCACCTCAAGTCCTACTA | XM_021567440.1 |
| MX1-R | CGGATCACCATGGGAATCTGA |  |
| LGP2-F | AGTTTGGCACGCAGGAGTA | ALE66118.1 |
| LGP2-R | CAAGCAGGAAGAAGTCGGT |  |
| TRIM25-F | AAAGATTCACCCCAAAACC | ACN11344.1 |
| TRIM25-R | AAGGCAGGGGAATCATAGT |  |
| RAG1-F | CAGAGGTACTACAGGAAATGG | XM_036933300.1 |
| RAG1-R | TTACTGGTCTTCAAGCAATG |  |
| IFNAR-F | CAGAGCCTCAGGAAGAACT | AGO14285.1 |
| IFNAR-R | CAAGGGGTAGAAGAGCATA |  |
| MDA5-F | CAGTGGAGATGACGATGGG | NP_001182108.1 |
| MDA5-R | ACTTGGCGTTCTTGTGCTT |  |
| IL-1β-F | ACCGAGTTCAAGGACAAGGA | NM_001124347 |
| IL-1β-R | CATTCATCAGGACCCAGCAC |  |
| IL-6-F | GCTCGGGACAGACGGTGAAA | NM_001124657 |
| IL-6-R | GAAACTCCTCCACAAACTGTTG |  |
| IL-8-F | GGCCCTCCTGACCATTACTGA | NM_001124362 |
| IL-8-R | TCCAGACAAATCTCCTGACCG |  |
| IL-10a-F | CGAAGGGATTCTACACCACTTG | NM_001245099 |
| IL-10a-R | GAGATTTAAAGTCGTTGTTGTTCTG |  |
| IL-10b-F | GAGCTGGAGACACCTCTTCTC | NM_001246350 |
| IL-10b-R | TAAAGTTGTGTGTTCCATTGTTG |  |
| TNFα1/2-F | ATGGAAGACYGKCAACGATGC | NM_001124357 |
| TNFα1/2-R | CGTCATCCTTTCTCCACTGCAC | NM_001124374 |
| TGFβ1a-F | GCACACGTACATACCGTCCA | XM_021591332 |
| TGFβ1a-R | GCTTACTGGTGTTCTCACTTTG |  |
| IL-17A/F1a-F | CGAAAGGAATGAAGGTGACAAA | NM_001124619 |
| IL-17A/F1a-R | TCTCCACTGTAGTGCTTTTCCA |  |
| Cath1-F | GAACACTGACCTGTCTACATTTG | NM_001124480 |
| Cath1-R | GACACAATTTTTGCCTCTGGA |  |
| Cath2-F | CATGGAGGCAGAAGTTCAGAAGA | KP347625 |
| Cath2-R | GAGCCAAACCCAGGACGAGA |  |
| NOD2a-F | GCTGTAGCAAACTGGCTCAAA | HM133906 |
| NOD2a-R | ATGCCCCAGGACAGGAGTAG |  |
| NOD2b-F | TCTCCCGCTGAAACCTGGTC | HM133907 |
| NOD2b-R | ACCCTCAGAAGAGCCACCAC |  |
| Lyz2-F | AGCCGCTACTGGTGTGACGA | NM_001124716 |
| Lyz2-R | CTGACAGTGAAGGCGCCAAG |  |
| Muc5ac-F | CCCTGAGTCTTCGGTTTA | XM_021573678 |
| Muc5ac-R | GCTGCCCTGTAGGATGTT |  |
| Muc2-F | GGAATCGTGGTGGACATG | XM_036968563 |
| Muc2-R | AGGCTAGGTAGCGAAAGG |  |

Table S2 The scoring system for the pathology findings in GB.

| Criterion | Qualitative description | Score |
| --- | --- | --- |
| Amount of inflammatory cell in mucosal surface per mucosal fold | 0-2 | 0 |
|  | 3-7 | 1 |
|  | ≥8 | 2 |
| Amount of cell with excessive mucoid secretion per mucosal fold | 0-1 | 0 |
|  | 2-4 | 1 |
|  | ≥5 | 2 |
| Degree of disrupted mucosal  epithelium | Minimal | 0 |
|  | Moderate | 1 |
|  | Marked | 2 |
| Degree of epithelium reaction to AB-PAS | Minimal | 0 |
|  | Moderate | 1 |
|  | Marked | 2 |

Table S3 Reads information of the 16S rRNA sequencing.

| Sample | Input | Filtered | Merged |
| --- | --- | --- | --- |
| GB_C1 | 141997 | 123234 | 91509 |
| GB_C2 | 136608 | 119166 | 96414 |
| GB_C3 | 137396 | 118806 | 93671 |
| GB_C4 | 141213 | 123587 | 99227 |
| GB_4DPI_1 | 137180 | 104884 | 67554 |
| GB_4DPI_2 | 134710 | 109820 | 84117 |
| GB_4DPI_3 | 139504 | 115640 | 93625 |
| GB_4DPI_4 | 135491 | 108061 | 85620 |
| GB_28DPI_1 | 136624 | 118379 | 91753 |
| GB_28DPI_2 | 135541 | 119334 | 99002 |
| GB_28DPI_3 | 138991 | 117391 | 86197 |
| GB_28DPI_4 | 135577 | 109904 | 80761 |
| Bile_C1 | 145736 | 129044 | 107406 |
| Bile_C2 | 135295 | 119552 | 101687 |
| Bile_C3 | 147966 | 130246 | 110400 |
| Bile_C4 | 141900 | 124039 | 105071 |
| Bile_4DPI_1 | 133172 | 116131 | 96586 |
| Bile_4DPI_2 | 134657 | 118598 | 100131 |
| Bile_4DPI_3 | 139389 | 120771 | 97511 |
| Bile_4DPI_4 | 144426 | 123547 | 97464 |
| Bile_28DPI_1 | 147542 | 130078 | 110843 |
| Bile_28DPI_2 | 135062 | 120032 | 102647 |
| Bile_28DPI_3 | 138355 | 110110 | 76721 |
| Bile_28DPI_4 | 143898 | 125296 | 106097 |
| FM_C1 | 133656 | 115427 | 94131 |
| FM_C2 | 139515 | 122478 | 112259 |
| FM_C3 | 146080 | 128327 | 110943 |
| FM_C4 | 146135 | 118188 | 106981 |
| FM_4DPI_1 | 132755 | 116993 | 102409 |
| FM_4DPI_2 | 147780 | 122922 | 101469 |
| FM_4DPI_3 | 135763 | 119064 | 101880 |
| FM_4DPI_4 | 138079 | 118811 | 98087 |
| FM_28DPI_1 | 144799 | 127543 | 108869 |
| FM_28DPI_2 | 148026 | 123488 | 100290 |
| FM_28DPI_3 | 137411 | 120732 | 103850 |
| FM_28DPI_4 | 138087 | 101030 | 71527 |
| MM_C1 | 142022 | 119673 | 93353 |
| MM_C2 | 138738 | 121807 | 105848 |
| MM_C3 | 134747 | 116313 | 100165 |
| MM_C4 | 135037 | 118408 | 101420 |
| MM_4DPI_1 | 112213 | 82948 | 69912 |
| MM_4DPI_2 | 88864 | 75110 | 73457 |
| MM_4DPI_3 | 84952 | 72283 | 69627 |
| MM_4DPI_4 | 127383 | 95154 | 82696 |
| MM_28DPI_1 | 140173 | 110680 | 83163 |
| MM_28DPI_2 | 141491 | 104004 | 79624 |
| MM_28DPI_3 | 140145 | 109267 | 85132 |
| MM_28DPI_4 | 132321 | 106211 | 87145 |
| HM_C1 | 145684 | 128344 | 109566 |
| HM_C2 | 143559 | 125579 | 103646 |
| HM_C3 | 137395 | 117387 | 94325 |
| HM_C4 | 140726 | 122958 | 100663 |
| HM_4DPI_1 | 144223 | 114959 | 89030 |
| HM_4DPI_2 | 144912 | 103283 | 76037 |
| HM_4DPI_3 | 135824 | 98592 | 72935 |
| HM_4DPI_4 | 138611 | 104172 | 80930 |
| HM_28DPI_1 | 134735 | 119611 | 97661 |
| HM_28DPI_2 | 133809 | 117841 | 95894 |
| HM_28DPI_3 | 133592 | 117268 | 97167 |
| HM_28DPI_4 | 141707 | 124063 | 100534 |
